# Non-Targeted Detection of Adulterants in Almond Powder Using Spectroscopic Techniques Combined with Chemometrics

**DOI:** 10.3390/foods9070876

**Published:** 2020-07-03

**Authors:** Mohammad Akbar Faqeerzada, Santosh Lohumi, Rahul Joshi, Moon S. Kim, Insuck Baek, Byoung-Kwan Cho

**Affiliations:** 1 Department of Biosystems Machinery Engineering, College of Agriculture and Life Science, Chungnam National University, Daejeon 34134, Korea; akbar.faqeerzada@gmail.com (M.A.F.); santosh.sanny123@gmail.com (S.L.); rahul.joshi98@yahoo.com (R.J.); 2 Environmental Microbial and Food Safety Laboratory, Agricultural Research Service, United States Department of Agriculture, Powder Mill Road, BARC-East, Bldg 303, Beltsville, MD 20705, USA; Moon.Kim@ars.usda.gov (M.S.K.); Insuck.baek@ars.usda.gov (I.B.); 3 Department of Smart Agriculture System, College of Agricultural and Life Science, Chungnam National University, Daejeon 34134, Korea

**Keywords:** food adulteration, almond powder, nondestructive test, non-targeted detection, one-class classification, FT-IR and FT-NIR spectroscopy

## Abstract

Methods that combine targeted techniques and chemometrics for analyzing food authenticity can only facilitate the detection of predefined or known adulterants, while unknown adulterants cannot be detected using such methods. Therefore, the non-targeted detection of adulterants in food products is currently in great demand. In this study, FT-IR and FT-NIR spectroscopic techniques were used in combination with non-targeted chemometric approaches, such as one-class partial least squares (OCPLS) and data-driven soft independent modeling of class analogy (DD-SIMCA), to detect adulterants in almond powder adulterated with apricot and peanut powders. The reflectance spectra of 100 pure almond powder samples from two different varieties (50 each) were collected to develop a calibration model based on each spectroscopic technique; each model was then evaluated for four independent sets of two varieties of almond powder samples adulterated with different concentrations of apricot and peanut powders. Classification using both techniques was highly sensitive, the OCPLS approach yielded 90–100% accuracy in different varieties of samples with both spectroscopic techniques, and the DD-SIMCA approach achieved the highest accuracy of 100% when used in combination with FT-IR in all validation sets. Moreover, DD-SIMCA, combined with FT-NIR, achieved a detection accuracy between 91% and 100% for the different validation sets and the misclassified samples belong to the 5% and 7% adulteration sets. These results suggest that spectroscopic techniques, combined with one-class classifiers, can be used effectively in the high-throughput screening of potential adulterants in almond powder.

## 1. Introduction

Food authentication networks are expanding to meet the demands of determining the safety of supplemented food products, to assess whether a food product matches the package description and adheres to regulations, and to offer advanced, non-destructive technologies. In recent years, the trend of adulterated high-value powdered food products has risen as a result of production practices that encourage and enable the illegal gain of profits. For instance, almond is considered the most nutritious nut and is consumed globally in different forms, such as in processed food, and particularly in bakery and confectionery products [1]. Owing to its high price and high global consumption, almond powder has become highly susceptible to economic adulteration with powders of less expensive nuts. Apricot powder is the most typical adulterant in almond powder, and is used because it has a lower price and similar color, texture, odor, and other physical characteristics compared to almond [2]. Peanuts are the second most commonly used almond adulterant globally; they have a substantially similar chemical composition and a considerably lower price. Although apricots do not exert detrimental effects on human health, they reduce the nutritional value of the product [3]. Conversely, peanut adulteration in almond powder can be life-threatening to individuals with peanut allergy. Therefore, it is essential to develop promising tools and techniques for determining the purity of almond powder.

Spectroscopic techniques coupled with chemometrics are known to be reliable tools for non-destructive chemical detection based on the accuracy and sensitivity of instruments. The most commonly used non-destructive tools, in combination with chemometric methods, are employed as targeted techniques that focus only on detecting materials of a known class or those that were added to the data set during the development of the calibration model. Recently, spectroscopic techniques, such as near-infrared (NIR), infrared (IR), and Raman spectroscopy, coupled with different chemometric techniques, have been used for the detection of adulterants in powdered foods. Adulteration of onion powder with corn-starch was identified using Fourier transform near-infrared (FT-NIR) and Fourier transform infrared (FT-IR) spectroscopy [4]. The quantitative analysis of Sudan dye adulteration in paprika powder using FT-IR spectroscopy [5], the detection of metanil yellow in turmeric using FT-Raman and FT-IR spectroscopy [6], and the determination of adulterants and contaminants in milk powder using NIR spectroscopy [7] have also been reported. These studies targeted specific representative compounds for specific adulterants; however, unknown adulterants will evade detection when such targeted techniques are used. The development of non-targeted analytical tools is essential for validating food authenticity to ensure consumer health and to increase confidence in product purchase. To facilitate the same, there has been a shift from target analysis to non-targeted detection that has gained popularity in food authenticity analysis. For instance, the application of the one-class partial least squares (OCPLS) approach in NIR spectral data analysis for classifying the geographical origin of Chinese herbs yielded excellent results [8]. Another study demonstrated the potential of the data-driven soft independent modeling of class analogy (DD-SIMCA) approach in the non-targeted detection of wheat flour and cassava adulterants in acai (*Euterpe oleracea*) [9].

In this study, we investigated the feasibility of using FT-IR and FT-NIR spectroscopic techniques combined with one-class classification (OCC) approaches for the non-targeted detection of adulterants in almond powder. DD-SIMCA and OCPLS are class modeling techniques that only model the target class and determine the threshold boundaries during model development. These types of OCC methods appear to be more effective than previously mentioned conventional classification techniques, particularly in adulteration analysis, because the acceptance boundaries for pure samples can be defined. Previous studies have compared OCC approaches with different chemometric techniques for food adulteration analysis, and highlight the significant potential of one-class modeling for the non-targeted detection of food adulterants [10]. Recent studies, such as that on the non-targeted detection of melamine in milk powder [11], have displayed a preferential shift from targeted to non-targeted analytical techniques. The non-targeted detection of native eggs by class modeling [12] and the authentication of juice samples from an antioxidant and chemical perspective [13] were performed in certain studies. Food adulteration analysis is not the only application that employs class modeling for analyzing the obtained data using a variety of approaches and obtaining reliable results. These techniques also yield remarkable results in quality analysis of pharmaceuticals—there have been studies on the determination of the active pharmaceutical ingredient content [14] and the active pharmaceutical ingredient content in the intact uncoated tablets of loratadine [15].

The non-targeted detection techniques are majorly used for the analysis of measurement data derived using spectroscopic techniques, which are reliable for large and small quantities of samples where the application of the technique is unaffected by the number of data. Moreover, the collected spectral data do not present the exact same pattern if the samples are heterogeneous, as in the case of adulterated powdered food samples. Such dissimilarities between two spectra of the same samples measured at different time points can significantly influence the classification and prediction performance of conventional multivariate analysis methods. Therefore, the present study adopted a combined approach of non-targeted detection analysis techniques, using DD-SIMCA and OCPLS to process FT-IR and FT-NIR data for the detection of unknown food adulterants in two different varieties of almond powders adulterated with ground apricots and peanuts. Although the adulterants were known, this information was not used to evaluate the performance of the non-targeted methods in the analysis of almond powder samples containing unknown adulterants.

This study primarily aimed to develop a non-targeted detection model based on FT-IR and FT-NIR spectroscopic techniques for the detection of unknown adulterants in almond powder samples. The spectral analysis of two different varieties of almond powder adulterated with apricot and peanut powders was conducted to demonstrate that this methodology can effectively detect various kinds of potential adulterants even in samples of different varieties.

## 2. Materials and Methods 

### 2.1. Sample Preparation 

The experimental samples comprised two different varieties of almond powders purchased from a commercial manufacturer, and apricot kernel powder purchased from an authorized food production company (Agriculture Corporation Neulgreen) in Daejeon, South Korea. Peanuts were purchased from the local supermarket in the same city; the pods and seed coats were removed manually, and the nuts were powdered using a commercial grinder. The samples were maintained at room temperature (20–22 °C) until further use. Variation in sample particle size generates noise and other artifacts in the extracted spectral data [16]; therefore, to obtain similar-sized particles of almond powder and adulterant materials, all samples were first sieved using a 250 μm mesh sieve. The almond powder samples were adulterated with 11 different concentrations of apricot powder (0–50%, with 5% increments) with 10 replicates. The second variety of almonds was adulterated in the same manner as the first variety with adulterant concentrations of 0%, 7%, 15%, 22%, and 30% (10 samples of 0% and 10 samples in each adulterated group). Sample information for the pure and adulterated ones used for the calibration and validation data sets is given in Table 1. One hundred pure samples from the two varieties of almonds were used to develop a model for both FT-IR and FT-NIR techniques, and in every two sets of validation, a total of 110 samples were used for the validation of the first variety of almond. The second validation set consisted of 50 samples (40 adulterated and 10 pure) for the second variety of almond. 

The adulterated sample was 7 g for all concentrations. A mixture of almond and peanut powder was prepared in the same manner as that followed for the almond and apricot powder mixture. In addition to the adulterated samples, samples of pure apricot powder and peanut powder were also prepared to examine their spectral features. Each combination was mixed manually and then transferred to a snap-cap vial. Further mixing was accomplished by placing the filled vials on a high-speed shaker (Vortex-Genie 2, Scientific Industries, Inc., model G560, Bohemia, NY, USA) for 1 min.

### 2.2. Spectral Collection 

The NIR reflectance spectra of the pure and adulterated almond powder samples were obtained using an FT-NIR spectrometer (Antaris II FT-NIR analyzer, Thermo Fisher Scientific Co., Waltham, MA, USA) equipped with an InGaAs detector. First, the sample holder was furnished with an accessory that contained a central hole (20 mm thickness × 20 mm diameter) to maintain uniformity in the shape and thickness of the sample over the irradiated surface during spectral collection. The reference calibration was measured with a golden slit before each sample scan was obtained. The instrument was set at diffuse reflectance, and 32 successive scans were obtained for every single sample in the range of 4000–10,000 cm^–1^ (1000–2500 nm) at a 4 cm^–1^ interval. The averaged spectra were collected for analysis. 

To obtain the FT-IR spectral data in the mid-IR (MIR) region (4000–650 cm^–1^), a Nicolet 6700 (Thermo Fisher Scientific Co., Waltham, MA, USA) FT-IR spectrophotometer configured for attenuated total reflectance sampling and having a deuterated triglycine sulfate detector and a KBR beam splitter controlled by the OMNIC software was used. For scanning, the sample was placed on the diamond crystal sampling plate and clamped with a pointed tip. The background data was collected before scanning for spectral collection using a free sample on the crystal. Additionally, for each scan, the attenuated total reflectance crystal, and pointed tip were cleaned to remove any remaining inferences for the sample underscan. The instrument was set to scan each sample with 32 scans at a 4 cm^–1^ interval, and the mean spectra of each sample was used for further analysis.

## 3. Data Analysis 

### 3.1. Spectral Preprocessing

**Figure 1:**
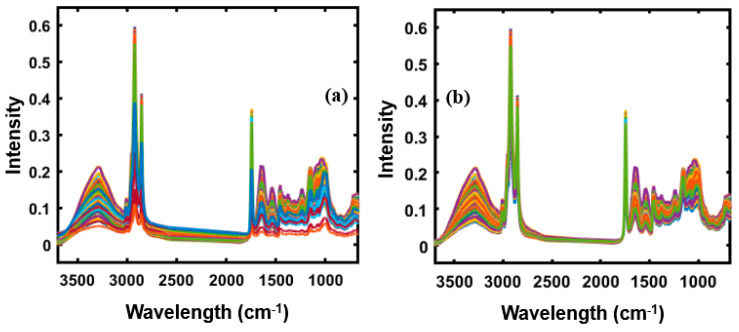
(**a**) Original FT-IR spectra of apricot-adulterated almond samples, and (**b**) the standard normal variate (SNV) preprocessed spectral data.

The raw spectral data obtained from the spectroscopic instruments contain systemic noise due to light scattering, baseline shift, instrumental drift, and slope variation caused by differences in particle size [17,18,19,20]. Therefore, pretreatment of the obtained raw data by appropriate mathematical analysis is important to enhance crucial information derivable from the sample and remove unwanted variations from the spectral data [21]. In this study, the normalization, smoothening, multiplicative scatter correction (MSC), standard normal variate (SNV), and Savitzky–Golay first and second derivative preprocessing methods were used to eliminate noise and undesired elements from the obtained spectral data for both spectroscopic techniques. Figure 1a,b represents the original FT-IR spectra and the SNV preprocessed spectral data of apricot-adulterated almond powder samples, respectively. The FT-NIR spectral data were also preprocessed before model development. The original spectra shown in Figure 1a was highly affected by the baseline shift and scattering effect; however, those effects were mitigated or removed by applying SNV preprocessing as shown in Figure 1b.

### 3.2. DD-SIMCA and OCPLS

The spectroscopy data comprise several variables, and interpreting data without the help of chemometric multivariate analytical methods and tools can be challenging. In this study, the DD-SIMCA and OCPLS multivariate analytical techniques were adopted and used as one-class classifier techniques.

First, the DD-SIMCA model was developed using a calibration set consisting of 100 pure spectral data from both varieties of almond powder, as explained in Section 2. The initial task was to determine the appropriate number of factors for each model. In this study, the appropriate number of factors was chosen by examining the sensitivity of the model. For this purpose, the resulting sensitivity was evaluated by adding 1–10 factors to the model. Two distances are crucial in the projection term-related technique: the score distance (SD, leverage) and the orthogonal distance (residual), calculated by the model. The adulterated and pure samples were separated by two borders or two levels of decision boundaries with the application of principal component analysis (PCA). The acceptance areas for the pure and adulterated samples are defined by the model based on the chi-square test. The developed calibration model was then tested for the detection of adulterants in two sets of external data for almonds and apricots and two sets for almonds and peanuts. A summary of the descriptive statistics for the calibration and validation data sets is presented in Table 1. New samples were assigned by adopting an alpha value of 0.01 for type I errors. The DD-SIMCA analysis was performed using a GUI toolbox developed using the MATLAB software [22].

Three parameters, namely sensitivity, specificity, and total classification accuracy, were used to determine the test performance of DD-SIMCA. The sensitivity indicates the number of true positive decisions (pure almond powder samples in this work)/(number of actual positive cases). The specificity indicates the number of true negatives (adulterated samples)/(number of actual negative samples). The total classification accuracy is the number of correctly detected samples/total number of samples.

OCPLS is based on the partial least squares (PLS) approach, while DD-SIMCA was based on PCA. OCPLS defines the distribution of data by measuring two distances. When an OCPLS model is built, two types of distance measures are used: Hotelling’s T^2^, based on the SD, and the absolute centered residual (ACR) of response. The latent variables (LVs) are calculated according to the cross-validation. SD is a measure of the distance from an object to the center of the class in the space covered by the primary OCPLS LVs. ACR is the dispersion measure of the projection onto the vector of OCPLS regression coefficients. An excessively high SD or ACR value indicates that an object has deviated from the bulk of the class. According to the values of ACR and T^2^, a test object can be assigned to one of the following four groups: regular or normal objects (with both small SD and ACR), good leverage objects (with a large SD and a small ACR), response outliers (with a small SD and a large ACR), and bad leverage objects (with both large SD and ACR); for more information about the toolbox, the original article can be referred to [23].

The parameters for quality analysis of the samples were calculated based on the calculated response by the model as a true positive (a positive response for a positive sample), false positive (a positive response for a negative sample), true negative (a negative response for a negative sample), and false negative (a negative response for a positive sample).

Sensitivity = (No. of detected pure samples/No. of total pure samples) ∗ 100 (1)

Specificity = (No. of detected adulterated samples/No. of total adulterated samples) ∗ 100 (2)

Additionally, the calibration model was used to construct a plot representing the number of samples that were characterized as regular, extreme, and outlier. Based on the number of extreme samples, the optimal number of factors were selected, such as the best number of factors to be fitted to our model with a low number of extreme samples, and the outliers were eliminated to develop a legitimate model.

## 4. Results and Discussion 

### 4.1. Spectral Profile of Almond Samples and Adulterants 

**Figure 2:**
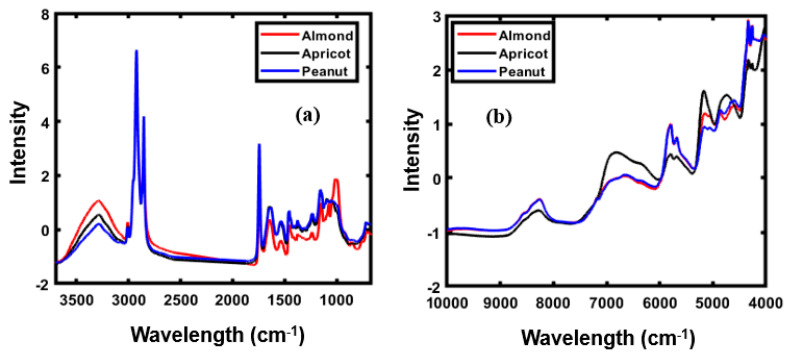
FT-IR (**a**) and FT-NIR (**b**) spectra of almond, apricot, and peanut.

Figure 2a,b presents the mean of the SNV-preprocessed spectral data for FT-IR and FT-NIR, respectively, revealing obvious differences in the absorption peak intensities. The FT-IR spectra was collected in the wave range of 600–4000 cm^−1^, and the spectral data before 3700 cm^−1^ were not considered for model development as information from this spectral region was irrelevant to this study. 

Figure 2a highlights the obvious differences in FT-IR peaks in the range of 1800–800 cm^–1^. As observable, both peanut and apricot generate similar spectral patterns, whereas the FT-IR spectral pattern of almond powder is notably different from those of apricot and peanut powders. In the functional group region (3600–1800 cm^–1^), the same spectral pattern is observed for all three powders except for the difference in peak intensity. The obtained spectra of nuts presented in Figure 2a primarily correspond to a combination of O−H stretching, C−H bending, and C−O stretching. Bands in the range of 3100–3000 cm^–1^ represent the stretching vibration of the C=CH cis-olefinic groups of unsaturated fatty acids present in almond [24,25,26]. 

The FT-IR spectrum has a broad range (3100–3810 cm^–1^), and almost the same spectral pattern for the three different powders, indicating the presence of CH_2_ functional group vibration stretching bands. Peaks in the range of 1850–1680 cm^–1^ correspond to the ester carbonyl functional group (C=O) of triacylglyceride esters [27,28]. The bands between 1600 and 1400 cm^–1^, particularly that at 1530 cm^−1^, correspond to the bending vibrations of the N−H functional group, primarily observed in amide I and amide II of the proteins present in almonds [29]. Peaks in the range of 1400–1200 cm^–1^, with several peaks at 1460–1230 cm^–1^, correspond the C−H bending vibrations in CH_2_ and CH_3_ [30]. The band detected approximately at 1045 cm^–1^ corresponds to the stretching vibration of C–O functional groups characteristic of the carbohydrate fraction of the samples [30]. 

The FT-NIR spectra of three different types of powdered samples, presented in Figure 2b, shows that the absorption intensities ranged from 5208 to 5050 cm^–1^, representing easily visualized peaks formed as a result of the O−H stretch and O−H band combination and the H−O−H deformation combination, corresponding to the starch content [31,32]. The spectral differences were observed from 7142–6250 cm^–1^, corresponding to the first overtone of the hydroxyl group. The precise position of these bands is extremely sensitive to hydrogen bonding in the starch molecule [33]. The most frequently visualized absorption bands appeared from 4000–7000 cm^–1^, formed approximately at 4536, 4987, 5174, 5376, and 6929 cm^–1^. The absorption band from 4536–4981 cm^–1^, assigned to the second overtone of the C−H functional group, also showed a small bump close at nearly 4744 cm^–1^. This absorption band is characteristic of the second overtone of the C−H stretching vibration of various functional groups, such as CH_2_ and −CH=CH− [34,35,36].

Similar bands corresponding to the functional C−H group were also observed in the 5174–5376 cm^–1^ NIR reflectance regions. In the absorption band spanning 6035–7458 cm^–1^, a broad bump centered at 6929 cm^–1^ was observed. This region corresponds to the first overtone of the C−H stretching vibration of the methyl and methylene groups, and a series of studies on this region of NIR spectra provided evidence of its correlation with proteins [34].

### 4.2. OCPLS-Based Non-Targeted Detection

OCC techniques (i.e., OCPLS and DD-SIMCA) were used to develop a robust calibration model for distinguishing pure powder samples from the adulterated ones. A robust calibration model was developed using data corresponding to 100 pure almond powder samples randomly selected from the two different varieties. The initial task was to determine the appropriate number of factors for the model by examining the sensitivity of the model as a function of the number of factors added to the model. Once the model was functional, we evaluated the model’s potential using an unknown set of objects not used in model construction. 

**Figure 3:**
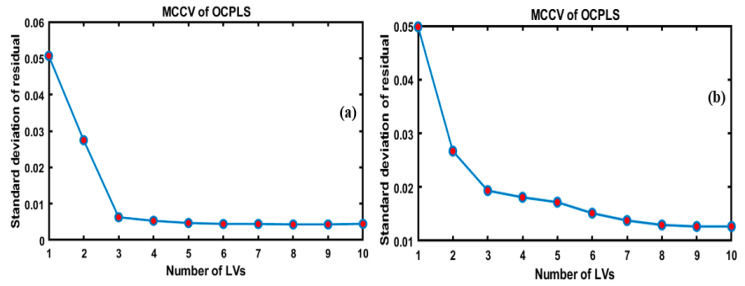
Standard deviation of residuals of one-class partial least squares (OCPLS) modeling vs. the number of latent variables in Monte Carlo cross-validation (MCCV) of OCPLS based on the FT-IR (**a**) and FT-NIR (**b**).

Initially, for the development of the OCPLS model using FT-IR and FT-NIR data, the Monte Carlo cross-validation (MCCV) was used to select the significant OCPLS components and estimate the standard deviation of the prediction residuals in the model. As shown in Figure 3a,b, the MCCV selected 10 components, and the lowest standard deviation of the cross-validation residual was obtained. Based on the figure, which presents a plot of the standard deviation against the number of LVs, the four LVs were adequate for preparing the desired model based on the FT-IR data used in Figure 3a, which depicts the plane graphs of LVS; however, increasing the number of LVs over eight increased the number of errors. In the same manner, the use of nine LVs yielded optimal results based on FT-NIR data, as shown in Figure 3b.

**Figure 4:**
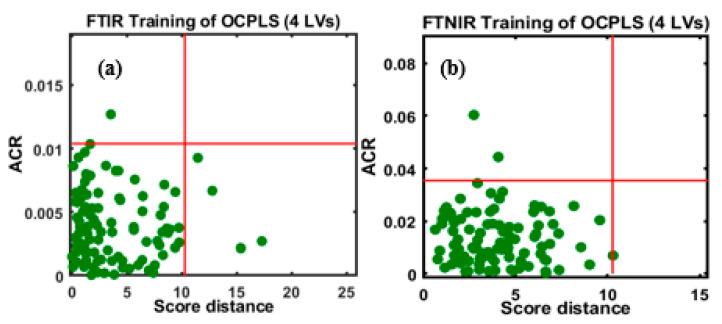
OCPLS-based calibration models for the (**a**) FT-IR and (**b**) FT-NIR data sets.

To mitigate any unwanted spectral variations caused by a range of factors, as mentioned previously, the spectral data were subjected to SNV transformation, and subsequently, the OCPLS calibration model was developed. Figure 4a,b depicts the models developed using the preprocessed FT-IR and FT-NIR spectral data, respectively, with assigned parameters. The two-dimensional (2-D) space model clearly indicates the distribution of samples in the 2-D space in both models.

Taking the ACR values into account, one sample was detected as an outlier with an ACR value over 0.01, while the remaining 99 samples were positioned at the bottom with low SD and ACR values, as shown in Figure 4a. The SD and ACR were defined as the acceptance area by the model. The SD and ACR values were fixed at 10.5 and 0.01, respectively; samples beyond the defined area indicated objects that deviated from the bulk of the class. The OCPLS outlier diagnosis depended on the ACR of the response variables and the OCPLS scores of primary LVs. 

The OCPLS model developed using the FT-NIR spectral data indicates that two samples were located outside the acceptance boundaries, and these were considered outliers (Figure 4b). The outliers were located beyond the red lines that limited the borders in the model displaying the upper confidence points for ACR and SD; the entire plane consisted of four regions; regular points with small SD and ACR (bottom left), good leverage points with large SD and a small ACR (bottom right), class outliers with small SD and a large ACR (top left), and bad leverage points with a large SD and ACR (top right). Therefore, to develop a robust and reliable calibration model, the detected outliers were excluded from both FT-IR and FT-NIR data sets, and new calibration models for each data set were developed.

**Figure 5:**
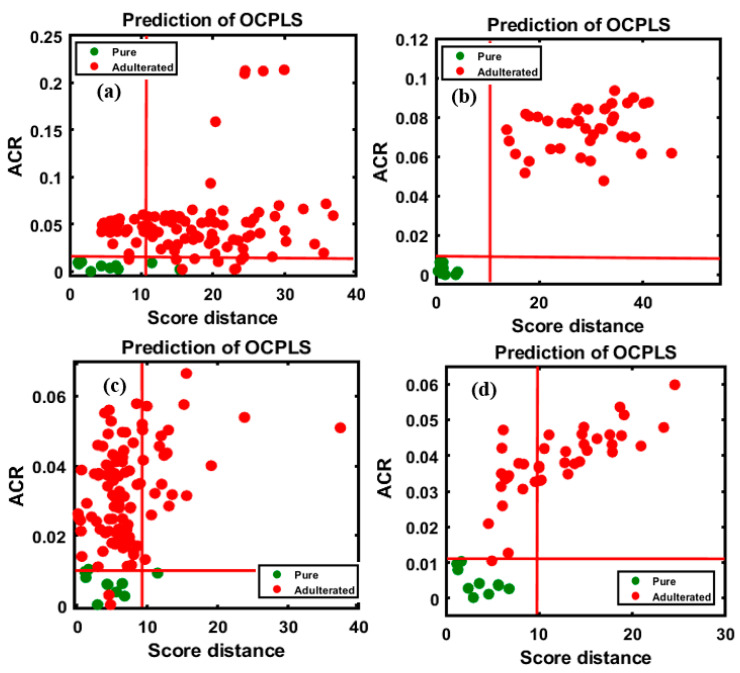
OCPLS-based classification plots for the four different FT-IR validation data sets: apricot-adulterated almond powder of the (**a**) 1st and (**b**) 2nd varieties, peanut-adulterated almond powder of the (**c**) 1st variety and (**d**) 2nd varieties.

The efficiency of the developed calibration model was then evaluated based on the classification of the four different validation sets, as mentioned in Section 2. The evaluation of model performances is presented in Figure 5a,b, which correspond to the first and second varieties of almond samples adulterated with apricot powder, respectively, and Figure 5c,d, corresponding to almond samples adulterated with peanut powder. Additionally, the establishment of an acceptance area was processed by dedicating the target and non-target sets of data in a group to check the performance of class modeling by the test set.

According to the model evaluation, the first validation set, corresponding to almond samples adulterated with apricot powder (Figure 5a), the classification had high sensitivity (100%) and specificity (8/10 pure, specificity of 80%); as observable from the figure, two samples from the pure almond group were located outside yet close to the acceptance area, possibly owing to minor changes in the spectral pattern. The total accuracy (98.18%) was calculated based on the total number of classified samples in the group. For the second variety (Figure 5b), 100% overall accuracy was achieved. As shown in Figure 5c, two peanut-adulterated almond samples were characterized as pure (98/100 adulterated samples); these misclassifications corresponded to the 5% concentration in the data set and this misclassification might have resulted from the similarity in the spectral pattern of almonds and peanuts. Among the pure samples (9/10 pure, specificity of 90%), a single sample was located outside the acceptance area. The total accuracy was 97.27% based on the correctly classified and misclassified samples. The results obtained for the second variety, as shown in Figure 5d, indicate 100% overall accuracy. Based on the results, the model performed almost similarly for both adulterants. The classification results are briefly summarized in Table 2, based on the degree of adulteration for each adulterant. 

**Figure 6:**
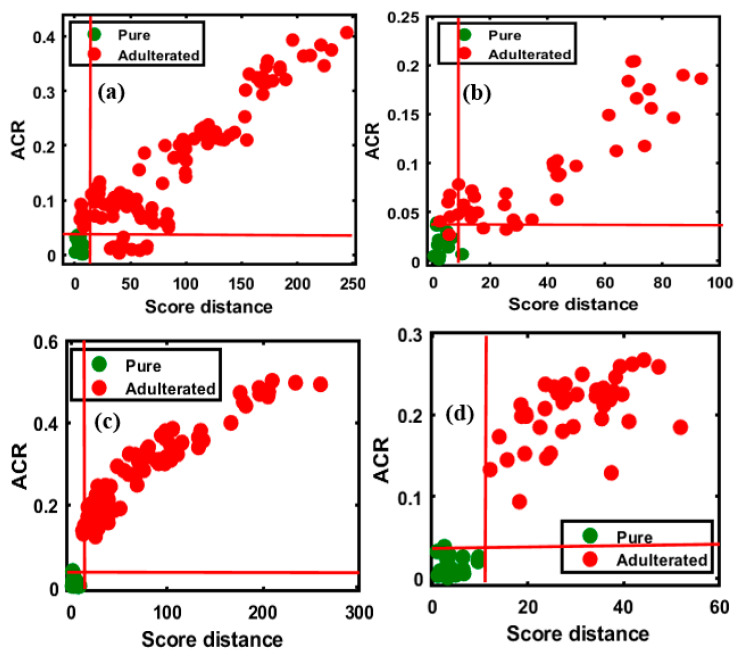
OCPLS-based classification plots for the four different FT-NIR validation data sets: apricot-adulterated almond powder of the (**a**) 1st and (**b**) 2nd varieties, peanut-adulterated almond powder of the (**c**) 1st and (**d**) 2nd varieties.

Similarly, the OCPLS model was used to evaluate the adulterated and pure samples of the same concentration percentages in the four sets of data based on the FT-NIR measurement. The results for apricot adulteration in the two varieties of almond are presented in Figure 6a,b, and those for peanut adulteration in the two varieties are presented in Figure 6c,d. The results of the OCPLS classification of the FT-NIR data are summarized in Table 3. 

Figure 6a depicts the high sensitivity of the model for the validation set (100/100 adulterated, sensitivity of 100%), while the specificity for the data set (14/15 pure samples, specificity of 93%) was marginally lower. A single pure sample was missing, possibly due to a minor change in the spectral pattern wherein a sample was recognized as an adulterant, because the sample located outside the acceptance range was almost within the pure sample area, and the overall accuracy was 99.13% for the validated set. With respect to the classification of the second group of adulterated samples, presented in Figure 6b, a lower accuracy was observed compared to the first group, with 97.50% sensitivity (39/40 adulterated samples), and a lower specificity (11/15 pure samples, specificity of 73.33%) was observed as well, possibly resulting from changes in the spectral pattern; the overall accuracy was 90.90%. The evaluation of the peanut-adulterated samples of the first variety, presented in Figure 6c, indicated high sensitivity (100%) and specificity (14/15 pure samples, specificity of 93%), with 99.13% overall accuracy. The evaluation of the peanut-adulterated samples of the second variety, presented in Figure 6d, indicated high sensitivity of 100%, while the specificity (14/15 pure samples, specificity of 93%) indicated a single misclassification; the overall accuracy was 98.18%. 

### 4.3. DD-SIMCA Model Based on FT-IR and FT-NIR Data

**Figure 7:**
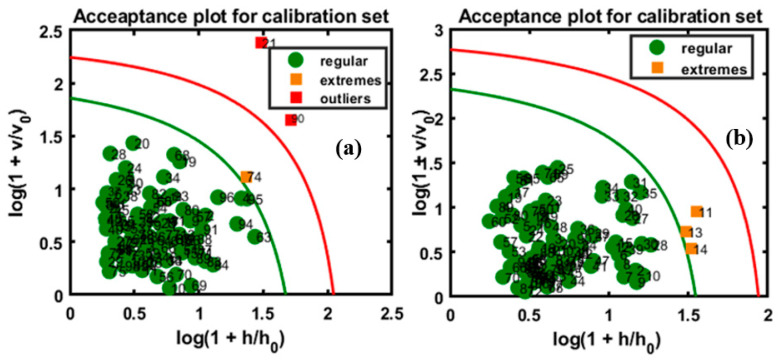
Calibration model based on FT-IR (**a**) and FT-NIR (**b**) data.

Based on the previous illustration of the DD-SIMCA algorithm, the pure samples were used to develop the models and the plots were constructed, as shown in Figure 7. Figure 7a,b represents the models constructed based on the FT-IR and FT-NIR data, respectively. After developing the model, the sensitivity was determined in the first step by adding a number of factors to the model and using a number of different principal components (PCs) until the best result was obtained. Eventually, four PCs were considered optimal. The misclassification of alien objects is a risk associated with OCC. It depends on the composition of the sample and might occur because the models are developed for the recognition of the target class and not the alien object. Therefore, to validate the model, it is necessary to use an independent set of target objects and to verify the model against a wide variety of alien objects. The model classification of the performance was evaluated with a new data set. The information on the new data sets and the obtained results are provided in Table 2 and Table 3. 

In class modeling, each class model can be represented by an acceptance area in the acceptance plot, defined by the orthogonal vs. score distance defined for a given α-value, which specifies the type I error as a share of the false negative decisions. Figure 7 shows the chi-square acceptance area of DD-SIMCA for the target class, where the green curve limits the acceptance area (α = 0.01), and the red curve limits the outlier area (γ = 0.01) for both models. In this plot, each sample of the training set was depicted by its position in the acceptance plot and was characterized either as a ‘regular’ sample, a sample from the target class, or as an ‘extreme’ sample. If a sample was located beyond the red line, it was considered an alien object. As shown in Figure 7a, the plotted model indicates that only one sample was an ‘extreme’ sample; the red line serves as the outlier border for presenting the γ-value, which segments the considered samples as outliers. According to the model, two samples were counted as outliers. Figure 7b represents the model which characterized three samples as ‘extreme’, which were present between the borders of the outlier and acceptance areas.

**Figure 8:**
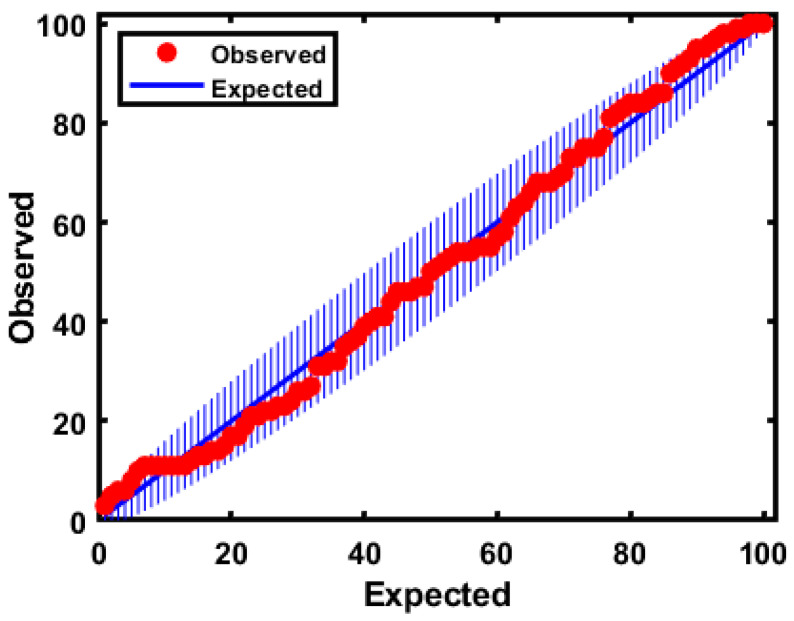
Extreme plot for the calibration set based on the model based on FT-IR.

Additionally, the model panel provided an extreme plot for observing the extreme samples against the theoretically predicted values. It indicated the number of data points in a straight line and the number of extreme and outlier samples in the plots were segmented from the vertical line. Moreover, the plot helped evaluate the performance of the class model when choosing the optimal number of PCs. As shown in Figure 8, owing to the consistency of the two numbers of samples outside the tolerance area and the congestion of the samples on the line, the number of samples were deviated slightly upward and did not clearly appear in the figure.

**Figure 9:**
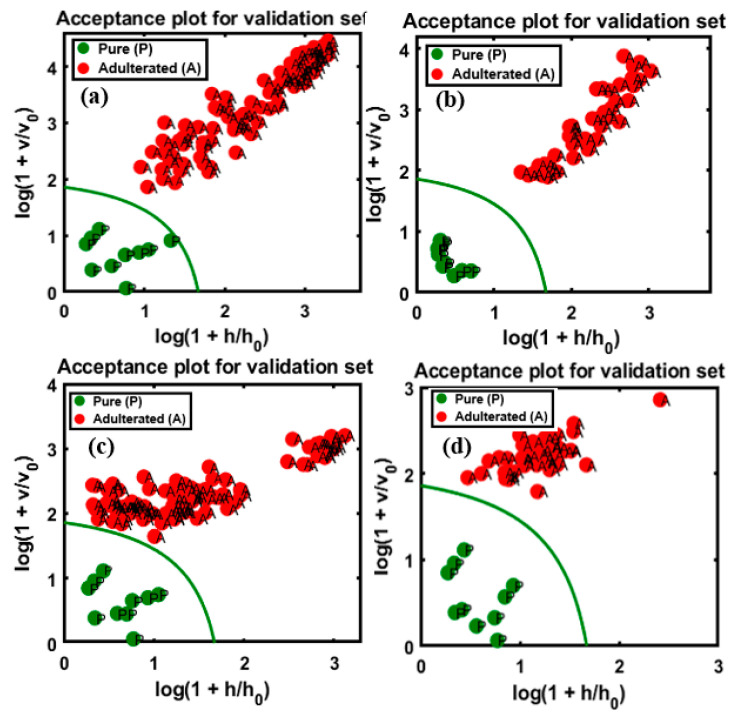
Prediction performance of the model with respect to the chi-square acceptance area of class modeling with pure (P) and apricot-adulterated (A) almond samples; the figures represent apricot-adulterated almond powder of (**a**) 1st and (**b**) 2nd varieties, peanut-adulterated almond powder of (**c**) 1st and (**d**) 2nd varieties.

The developed model validated the four sets of data, the plots for which are presented in Figure 9, and the total accuracy according to the above illustrations was calculated based on the sensitivity, which are summarized in Table 2, for all sets of data, including the training and test sets using both instruments. In the validation set, the performance of the developed model based on the FT-IR data for apricot-adulterated almonds of the first and second varieties are presented in Figure 9a,b, respectively. The predictive performance of DD-SIMCA for the chi-square acceptance area of the targeted class was denoted by “P” (pure) for the test set and by “A” for the adulterated samples. Figure 9c,d represents data for the peanut adulteration of the two almond varieties.

According to the figures, all the validated sets were well classified into the target and non-target sets of data. There is an observable clear and wide distance of acceptance between the target and non-targeted samples in Figure 9a,b,d, indicating lower chances of misclassification. Meanwhile, the target and non-targeted sets of data were placed within a close range in Figure 9c. Therefore, the four PCs used to develop the model cannot be reduced further. Based on the figures, all sets presented a high accuracy at 100%, according to the calculated sensitivity and specificity of the groups. This might be attributed to the sensitivity of the instrument used for the detection of the adulterated samples as well as to the application of DD-SIMCA, which separates target from non-targeted sets. According to previous reports, the spectral data from the MIR region are commonly used for structural identification (fingerprinting) of organic compounds because the absorption bands are representative of fundamental vibrations of a specific functional group [37], and this is the major advantage offered by MIR over NIR instruments with respect to the identification of trace elements. 

**Figure 10:**
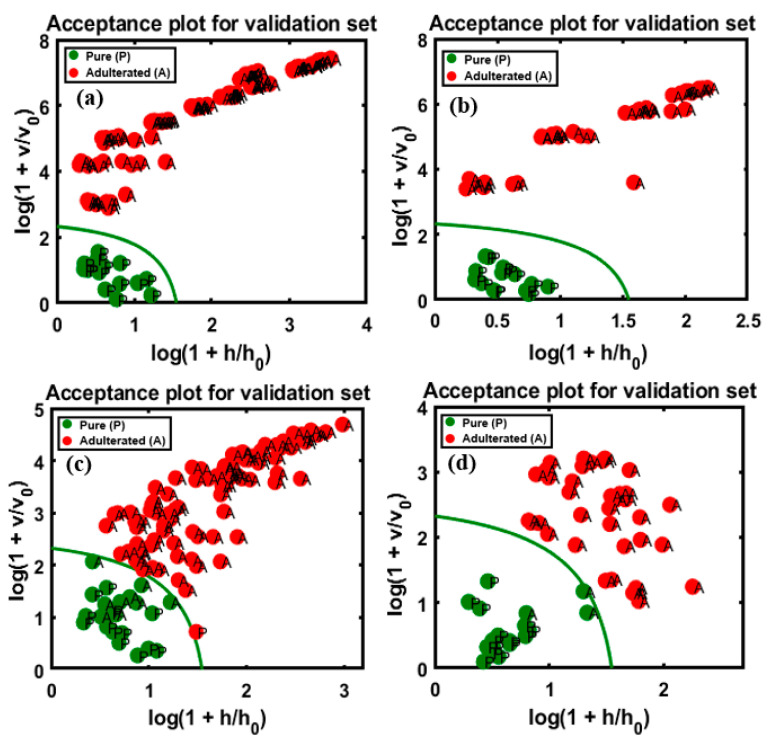
Predictive performance of the model based on the chi-square test determined by applying the DD-SIMCA method to the four different FT-NIR validation data sets: apricot-adulterated almond powder of the (**a**) 1st and (**b**) 2nd varieties, peanut-adulterated almond powder of the (**c**) 1st and (**d**) 2nd varieties.

The validated results based on the FT-NIR extracted spectral data was marginally less accurate than those based on the FT-IR data (present in Figure 10), because the predictive performance of DD-SIMCA for the chi-square acceptance area of the target and non-targeted classes was defined by the model with four PCs. The validation set of data are presented as follow: (a) and (b), representing apricot adulteration in the two varieties of almonds, and (c) and (d), representing peanut adulteration in the two varieties of almonds, respectively. The obtained results are briefly summarized in Table 3, calculated based on the sensitivity and specificity responses of the validated groups determined by the model.

As observable from Figure 10a,b, the sensitivity (100%) and specificity (100%) were highly accurate, as in the validated data set, and the acceptance area indicates the wide gap between the target and non-targeted data and the 100% overall accuracy of validation. Figure 10c indicates the low accuracy compared to the other validation sets, with lower specificity (14/15 pure samples, specificity of 93%) and sensitivity (91/100 adulterated samples, sensitivity of 91%) of the targeted and non-targeted data. A single sample was misclassified in the target class, while nine samples were misclassified in the non-targeted class, which corresponds to 5% concentration of the data on adulterated samples. Therefore, the total calculated accuracy was 90%. The second validation set, represented by Figure 10d, had a marginally higher accuracy, with 92% sensitivity (37/40 adulterated samples) and 100% specificity obtained for the data set. The three misclassifications corresponded to 7% concentration and the total calculated accuracy based on the presented sensitivity and selectivity of data was 94.54%. This could be attributed to the absence of sensitivity of the instrument at low adulteration percentages, which limits the application of the instrument. Earlier studies also reported this limitation of FT-NIR spectroscopy and stated that it had less than 10% adulteration detection rate. The adulteration of raspberry with apple could be detected at levels >10% using the PLS-NIR models [38]. The adulteration in *Echinacea* sp., could also be detected using FT-NIR spectroscopy provided the adulteration percentage was at least 10% [39]. The NIR bands are representative of the complex vibrational motion of chemical bonds that tend to deviate from harmonicity. These deviations result in bands shifting to higher energy levels (12,500–4000 cm^−1^) of fundamental vibrations, leading to a reduction in the NIR absorption intensity with the increase in the rank of overtones. Additionally, the NIR spectra include combination modes from the interaction of two or more vibrations occurring simultaneously in response to the absorption of single photon [40]. The structural selectivity of NIR for the positioning and combination of different overtones is relatively lower than that of MIR, and the NIR spectra has bands that are 10–100 times less intense than the corresponding fundamental bands in the MIR spectra [41]. This limitation could account for the misclassification of certain adulterated samples when the FT-NIR technique was used at 7% apricot adulterant concentration. 

Table 2 presents the results obtained from the OCPLS and DD-SIMCA validated models for each of the four sets of data. The adulterant columns refer to the validation group of samples considered (almond, apricot, and peanuts) in the two varieties. As explained, the total accuracy was calculated based on the sensitivity and specificity of the group by target and non-targeted sets of data.

Both techniques yielded encouraging results, as the performance values (sensitivity and selectivity) for the target and non-targeted groups were comparable. OCPLS yielded a minimum accuracy of 97.27% for peanut-adulterated almonds of the first variety and a maximum accuracy of 100% for the peanut-adulterated almonds of the second variety. The model showed similar accuracy for the apricot-adulterated almonds, with 98.18% and 100% accuracy for the two varieties, respectively. In comparison, the DD-SIMCA model performed better than the OCPLS model, as the sensitivity and specificity were calculated with 100% accuracy in all groups. In brief, the DD-SIMCA model yielded perfect results based on the FT-IR data, followed closely by the results of the OCPLS model.

Table 3 presents the results obtained using the OCPLS and DD-SIMCA models on the FT-NIR data.

Based on data presented in Table 3, the highest accuracy (99.13%) was achieved using OCPLS for the data on the first variety of apricot-adulterated almonds, while the lowest accuracy (90.90%) corresponded to the second variety of apricot-adulterated almonds. The OCPLS model yielded encouraging results for the peanut-adulterated almonds of both varieties. The DD-SIMCA model yielded results contrasting those obtained using the OCPLS model, with a higher accuracy of 100% for both groups of apricot-adulterated almonds, and an accuracy of 91.30% and 94.54% for the peanut-adulterated almonds of the first and second varieties, respectively. Comparatively, both groups presented almost similar results with minor differences in the accuracy achieved using the OCPLS model.

According to the results discussed earlier (based on the plots for the validation sets of data) and in Table 2 and Table 3, both instruments displayed sufficient sensitivity for the non-targeted detection of adulterated almond samples when used in combination with the OCC techniques (OCPLS and DD-SIMCA). According to the results of the validated sets based on the FT-IR data, the OCPLS model, with a minimum accuracy of 97%, had efficiency comparable to the DD-SIMCA model, which presented 100% accuracy for the validation sets. Meanwhile, based on the results obtained using the FT-NIR extracted data, both non-targeted techniques yielded similar results in terms of overall accuracy; however, the results differed according to the variety. As discussed previously, the misclassification of samples in each group could be explained with details related to each validation set, and these misclassifications mostly corresponded to 5% or 7% sample concentration. According to the FT-NIR spectral data, minor changes in the pure samples resulted in the model classifying them as adulterated samples, which can be explained by their proximity to the borderline in the acceptance area. This might result from the relatively lower structural selectivity for the positioning and combination of different overtones in NIR than that in MIR; therefore, according to results, the examination of the MIR is more effective with respect to spectral identifications, owing to the presence of the fingerprint region. Additionally, the data presented in the tables also indicate the comparatively lower accuracy of FT-NIR compared to that of FT-IR for both non-targeted detection techniques, owing to the misclassification of samples having less than 10% adulteration. However, there were no misclassified samples in the validation sets related to other groups. Therefore, it indicates that the FT-NIR shows lower accuracy owing to the presence samples having less than 10% adulteration. Finally, the combination of FT-IR and one-class classifiers appeared to function perfectly, where the DD-SIMCA presented higher quality results compared to the OCPLS. Since the potential of the developed chemometric models was tested for two different varieties of almond powder samples, these classification models can be easily updated by adding the spectral features of different varieties, origin, and different storage times of almond powder to the calibration set. 

## 5. Conclusions

This study primarily aimed to develop non-targeted models based on spectroscopic techniques (FT-IR and FT-NIR) to assess the purity of almond powder. With respect to the detection of unknown adulterants in almonds, OCC chemometric techniques, such as OCPLS and DD-SIMCA, have a distinct advantage over other traditional techniques that use a single model to validate unknown sets of samples. The OCC techniques develop a model for one-class without thorough information regarding other classes. For this purpose, the models based on these techniques were potentially evaluated using four sets of data for the quality analysis of adulterated almonds. However, both classifier analysis techniques achieved high performance in the distinction of adulterated samples from pure samples. In comparison, the model using FT-NIR data, which has lower sensitivity for detection of adulterated samples with less than 10% adulteration, showed lower accuracy than that using FT-IR data. Owing to the fingerprint region, the MIR can be used to easily identify the structural compounds of organic materials, based on the absorption bands formed as a result of vibrations in the specific functional groups. Therefore, FT-IR spectroscopy combined with DD-SIMCA yielded excellent results with 100% accuracy in all validations sets, and was closely followed by the OCPLS model, which had a marginally lower accuracy. This study demonstrated the potential of spectroscopic techniques combined with one-class classifiers for the non-targeted detection of adulterants in powdered food products.

## Figures and Tables

**Table 1:** Summary of sample information for the calibration and validation data set for data-driven soft independent modeling of class analogy (DD-SIMCA) and one-class partial least squares (OCPLS) analyses (excluding the number of samples, all units in %) based on the FT-IR- and FT-NIR-measured samples.

DD-SIMCA and OCPLS		Number of Samples	Maximum	Minimum
FT-IR	Calibration	100	100	0
	Validation 1	110	50	0
	Validation 2	50	30	0
FT-NIR	Calibration	100	100	0
	Validation 1	110	50	0
	Validation 2	50	30	0

**Table 2:** Summary table of model performance obtained by applying OCPLS and DD-SIMCA to FT-IR data.

Adulterants		Sensitivity (%)	Number of Correctly Classified Samples/Total Number of Samples	Specificity (%)	Number of Correctly Classified Samples/Total Number of Samples	Accuracy (%)
**DD-SIMCA Result**	Val-1st variety (almond + apricot)	100	100/100	80	8/10	98.18
	Val-2nd variety (almond + apricot)	100	40/40	100	10/10	100
	Val-1st variety (almond + peanut)	98	98/100	90	9/10	97.27
	Val-12nd variety (almond + peanut)	100	40/40	100	10/10	100
**OCPLS Result**	Val-1st variety (almond + apricot)	100	100/100	100	10/10	100
	Val-2nd variety (almond + apricot)	100	100/100	100	10/10	100
	Val-1st variety (almond + peanut)	100	100/100	100	10/10	100
	Val-2nd variety (almond + peanut)	100	100/100	100	10/10	100

**Table 3:** Summary table of model performance obtained by applying OCPLS and DD-SIMCA to FT-NIR data.

Adulterants		Sensitivity (%)	Number of Correctly Classified Samples/Total Number of Samples	Specificity (%)	Number of Correctly Classified Samples/Total Number of Samples	Accuracy (%)
**DD-SIMCA Result**	Val-1st variety (almond + apricot)	100	100/100	93	14/15	99.13
	Val-2nd variety (almond + apricot)	97	39/40	93	1/15	90.90
	Val-1st variety (almond + peanut)	100	100/100	93	14/15	99.13
	Val-2nd variety (almond + peanut)	100	40/40	93	14/15	98.18
**OCPLS Result**	Val-1st variety (almond + apricot)	100	100/100	100	15/15	100
	Val-2nd variety (almond + apricot)	100	40/40	100	15/15	100
	Val-1st variety (almond + peanut)	91	91/100	93	14/15	91.30
	Val-2nd variety (almond + peanut)	92.5	37/40	100	15/15	94.54
